# Probabilistic independent component analysis of dynamic susceptibility contrast perfusion MRI in metastatic brain tumors

**DOI:** 10.1186/s40644-019-0201-0

**Published:** 2019-03-18

**Authors:** Ararat Chakhoyan, Catalina Raymond, Jason Chen, Jodi Goldman, Jingwen Yao, Tania B. Kaprealian, Nader Pouratian, Benjamin M. Ellingson

**Affiliations:** 1 UCLA Brain Tumor Imaging Laboratory (BTIL), Center for Computer Vision and Imaging Biomarkers, University of California, Los Angeles, Los Angeles, CA USA; 2 Departments of Radiological Sciences and Psychiatry, David Geffen School of Medicine, University of California, Los Angeles, 924 Westwood Blvd., Suite 615, Los Angeles, CA 90024 USA; 3 Department of Neurosurgery, David Geffen School of Medicine, University of California, Los Angeles, Los Angeles, CA USA; 4 Department of Microbiology, Immunology and Molecular Genetics, University of California, Los Angeles, Los Angeles, CA USA; 5 Department of Bioengineering, Henry Samueli School of Engineering and Applied Science, University of California Los Angeles, Los Angeles, CA USA; 6 Department of Radiation Oncology, David Geffen School of Medicine, University of California, Los Angeles, Los Angeles, CA USA; 7 Brain Research Institute, David Geffen School of Medicine, University of California, Los Angeles, Los Angeles, CA USA; 8 UCLA Neuro-Oncology Program, University of California, Los Angeles, Los Angeles, CA USA

**Keywords:** Brain metastasis, Diffusion, Perfusion, ICA, Biomarker

## Abstract

**Purpose:**

To identify clinically relevant magnetic resonance imaging (MRI) features of different types of metastatic brain lesions, including standard anatomical, diffusion weighted imaging (DWI) and dynamic susceptibility contrast (DSC) perfusion MRI.

**Methods:**

MRI imaging was retrospectively assessed on one hundred and fourteen (*N* = 114) brain metastases including breast (*n* = 27), non-small cell lung cancer (NSCLC, *n* = 43) and ‘other’ primary tumors (*n* = 44). Based on 114 patient’s MRI scans, a total of 346 individual contrast enhancing tumors were manually segmented. In addition to tumor volume, apparent diffusion coefficients (ADC) and relative cerebral blood volume (rCBV) measurements, an independent component analysis (ICA) was performed with raw DSC data in order to assess arterio-venous components and the volume of overlap (AVOL) relative to tumor volume, as well as time to peak (TTP) of T_2_* signal from each component.

**Results:**

Results suggests non-breast or non-NSCLC (‘other’) tumors had higher volume compare to breast and NSCLC patients (*p* = 0.0056 and *p* = 0.0003, respectively). No differences in median ADC or rCBV were observed across tumor types; however, breast and NSCLC tumors had a significantly higher “arterial” proportion of the tumor volume as indicated by ICA (*p* = 0.0062 and *p* = 0.0018, respectively), while a higher “venous” proportion were prominent in breast tumors compared with NSCLC (*p* = 0.0027) and ‘other’ lesions (*p* = 0.0011). The AVOL component was positively related to rCBV in all groups, but no correlation was found for arterial and venous components with respect to rCBV values. Median time to peak of arterial and venous components were 8.4 s and 12.6 s, respectively (*p* < 0.0001). No difference was found in arterial or venous TTP across groups.

**Conclusions:**

Advanced ICA-derived component analysis demonstrates perfusion differences between metastatic brain tumor types that were not observable with classical ADC and rCBV measurements. These results highlight the complex relationship between brain tumor vasculature characteristics and the site of primary tumor diagnosis.

**Electronic supplementary material:**

The online version of this article (10.1186/s40644-019-0201-0) contains supplementary material, which is available to authorized users.

## Introduction

Brain metastases are the most common type of intracranial neoplasm [1, 2]. The majority of brain metastases originate from primary cancers in the lung (40–50%), breast (15–25%) or melanoma (5–20%) [3, 4]. The brain metastatic cascade is schematized by tumor cell dissociation from the host organ, intravasation into the vasculature, migration along the vessels and adhesion to the capillary bed, and lastly extravasation into the brain tissue through blood brain barrier (BBB) [5]. Recent studies speculate that tumor can grow at a clinically detectable stage with vessel co-option [6], a non-angiogenic mechanism [7]. Such processes have been reported in non-small cell lung tumors (NSCLC) [8] and melanoma [9]. Interestingly, Valiente et al., recently demonstrated that metastatic tumors can survive and grow while adhering to capillaries [10], causing resistance to anti-angiogenic treatments [11]. Evidence also suggests significant differences in vascular density between breast and melanoma brain metastases that may be influenced by genetic factors, including the expression of CD105, a transforming growth factor (TGF)-beta receptor endoglin [12].

In clinical practice, patients were often screened with brain lesion and some potential unknown primary tumor located in the body. A robust and highly specific diagnosis is very important, however, conventional imaging approaches encounter limited specificity to differentiate and predict primary lesions. MRI has been long time used to assess tumor localization and burden. Moreover, specific functional features could be extracted by using DWI and DSC-MRI [13]. The DWI derived measurement of ADC has been shown to correlate with tissue cellularity in brain lesions [14] as well as in metastatic brain lesions [15]. From DSC-MRI, rCBV can be estimated and used as a marker for hypervascularization, as studies have suggested rCBV may reflect vascular morphometry [16]. Additionally, arterial, venous and overlap (AVOL) components present within tumor volume could be extracted from ICA, based on DSC signal [17]. As another surrogate assessment of vascularization reflecting differences in the dispersion or tortuosity of the vessels, TTP may be extracted from normalized R_2_* signal for both arterial and venous ICA components.

In this study, we investigated the potential differences in tumor burden, water diffusivity and perfusion features and that between different subtypes of secondary brain lesions. We additionally hypothesized possible differences between metastatic lesions, especially in the proportion of each vascular component (arterial, venous or overlap), present within contrast enhancing region.

## Methods

### Patient characteristics

Inclusion criteria were based on the known primary tumor location; specific secondary organ (brain parenchyma) and available brain MRI study including anatomical, diffusion- and perfusion-weighted images acquired prior to radiation treatment. After the discovery and histopathological diagnosis of primary neoplasm (breast, lung, etc), a whole body FDG-PET/CT was performed as part of active clinical surveillance to identify the presence of metastatic disease. If a metabolically active region was detected within the brain, it was assumed to be due to the primary tumor type, and a subsequent high-resolution brain MRI was performed. From August 2014 to December 2016, 114 patients (71 female and 43 male) were selected for the current retrospective study, including various primary tumors: breast (*n* = 27, female = 100%) lung adenocarcinoma (non-small cell lung cancer (NSCLC), *n* = 43, female = 51%) and ‘other’ (*n* = 44, female = 50%). The ‘other’ group, or non-breast and non-NSCLC tumors, consisted of different primary tumors (hepatocellular carcinoma, renal cell carcinoma, clear cell carcinoma-kidney, etc). More details of patient’s primary tumor location are reported for the former group (Additional file 1: Table S1). The median age of patents at time of imaging was breast = 56 years, NSCLC = 65 years and ‘other’ = 62.5 years.

### MRI acquisition

All images were acquired on either 1.5 T or 3 T scanners. T1-weighted images were acquired before and after contrast agent injection (gadopentetate-dimeglumine, Gd-DTPA, Magnevist) with repetition time (TR)/echo time (TE) ranging from 400 to 2100 ms/1.18–1.53 ms; slice thickness = 1–1.5 mm; number of slice = 118–192. DWI were acquired before injection of contrast with TR/TE = 4–13 s/65–124 ms; flip angle = 90 degree; slice thickness = 2-5 mm; matrix size = 128 × 128 and number of slices ranged 24–86. DSC perfusion MR images were acquired during contrast agent bolus with TR/TE = 1.1–2.4 s/17–45 ms; slice thickness = 4-5 mm; inter-slice gap = 4–6.5 mm; matrix size = 128 × 128; flip angle = 35^0^, 60^0^ or 90^0^; number of repetitions = 50–120 times for a total number of slices = 6–48. Gd-DTPA was power-injected through a venous catheter at standardized pre-load of 0.025 mmol/kg followed by a bolus dose of 0.075 mmol/kg.

### Post-processing

Contrast-enhanced digital T1 subtraction maps (delta T1 maps) were performed as previously described [18]. First, pre- and post-contrast T_1_-weighted images were registered and intensity normalized (NIMH MEG Core, Bethesda, MD; kurage.nimh.nih.gov/meglab/Med/3dNormalize) followed by voxel-by-voxel subtraction, resulting in ∆T1 maps highlighting areas of active tumor burden. ADC maps (expressed in μm^2^/ms) were computed offline using a mono-exponential model [19] using clinically available *b* values (either 0 and 1000s/mm^2^ or 0, 500 and 1000s/mm^2^).

Two types of analyses were performed with DSC perfusion data: 1) traditional estimation of rCBV and 2) estimation of vascular volume fraction of arterial and venous vasculature identified through ICA, using the MELODIC toolbox (FMRIB library).

For traditional perfusion measures, dynamic time series data were first motion corrected (MCFLIRT, FMRIB library). Next, rCBV maps were calculated using a bi-directional contrast agent leakage correction algorithm to model contrast flux into and out of the vasculature [20, 21]. Lastly, normalized rCBV (expressed in a.u.) was computed by dividing the rCBV map by the average rCBV value in within a 5 mm sphere in contralateral, normal appearing white matter (NAWM).

In order to estimate the volume fraction of “arterial” and “venous” vasculature within the tumor, independent component analyses (ICA) was applied to the dynamic time series data following motion correction [22]. Next, intensity normalization was performed over time frames and a single-session ICA model with 4 components was opted; arterial, venous [17] and structured noise for last two components (motion-related, physiological artifacts, sawtooth pattern, etc) [23] with a statistical threshold of *p* < 0.5 to preserve voxels with temporal patterns significantly different from noise. Results were visually inspected and arterial and venous components were selected according to presence of Circle of Willis (arterial) and major draining sinuses (venous). In questionable datasets the temporal arrival of the contrast bolus was considered, with components having the fastest arrival categorized as “arterial” and those with slower, delayed bolus arrival categorized as “venous”. A single investigator with ~ 5 years of experience initially categorized the components, which was confirmed by a second investigator with > 10 years of experience.

**Fig. 1:**
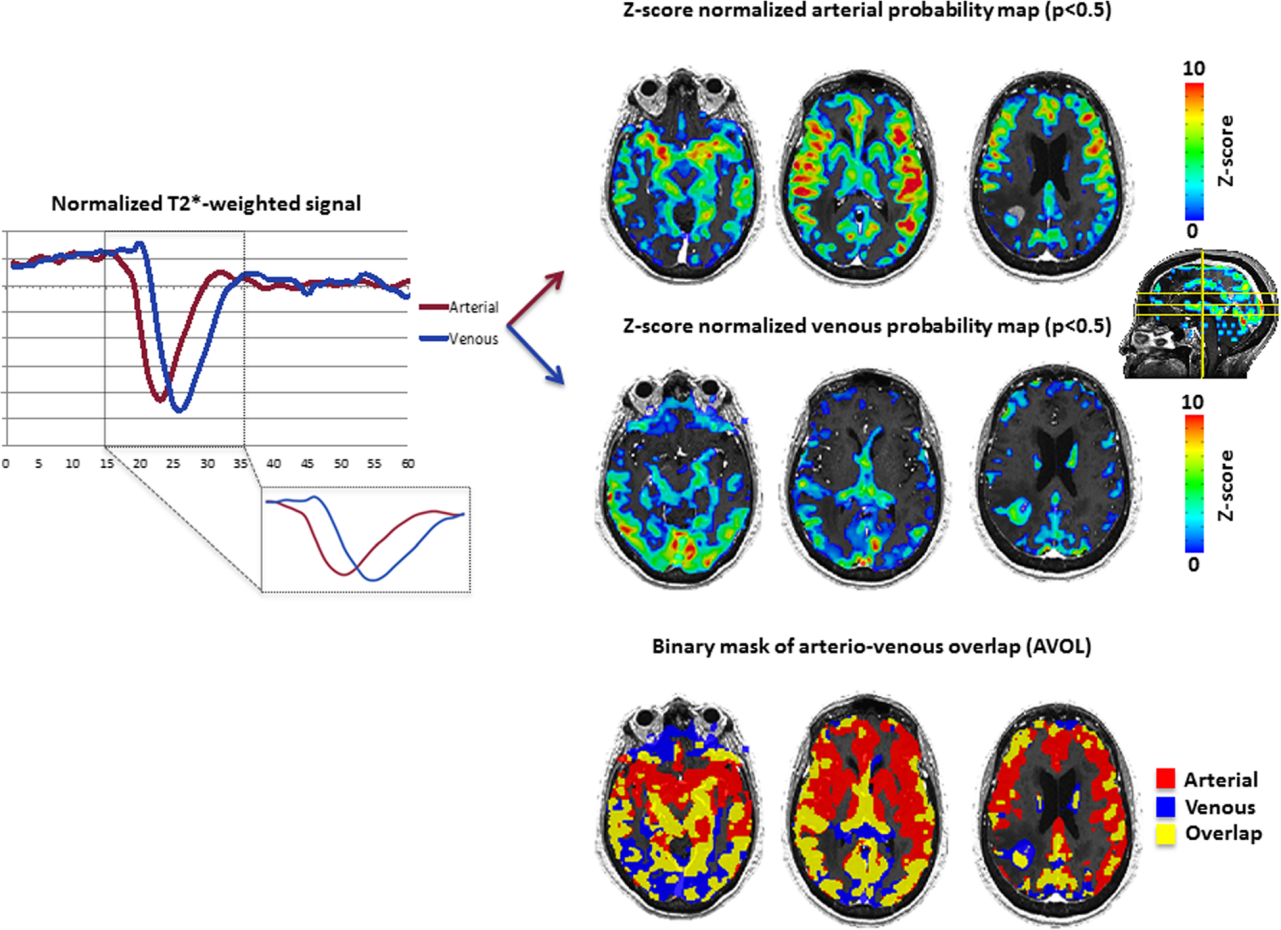
Independent component analysis (ICA) of dynamic susceptibility contrast (DSC-MRI). Left - Temporal evolution of normalized T_2_* from arterial and venous component. Right – ICA derived Z-score probability maps (*p* < 0.5) for respective components for one representative patient. Results are overlaid on anatomical T1-weigthed post-contrast image. Sagittal plane representing selected slices (from left to right) to cover polygon of Willis, sagittal sinus and tumor region. Binarized arterio-venous (positive Z-score values) and overlap mask (AVOL) are overlaid to the T1-weigthed post-contrast image

Z-score normalized maps were binarized (only positive voxels) to create an overlap mask that combine both arterial and venous mixed (AVOL map) regions (Fig. 1), similar to those described by LaViolette et al. [17]. Since ICA is highly dependent of bolus temporal evolution, we hypothesized existence of potential differences in TTP between arterial or venous components across the different tumor types. TTP has been calculated from the start of the inflection point of T_2_* signal. As we extracted TTP from normalized T_2_* after ICA processing, values are reported for each patient and not individual lesions.

### Image registration

Image registration was performed using FMRIB Software Library (FSL) linear image registration toolbox (FLIRT, http://www.fmrib.ox.ac.uk/fsl/; FSL Version 5.3). Perfusion and diffusion images were registered to high-resolution T_1_-weighted post-contrast image using a 12-degree of freedom transformation with a mutual-information cost function and a tri-linear interpolation.

### Regions of interest

Contrast-enhancing tumors (CET) from ∆T_1_ maps were segmented using a semi-automatic procedure as previously described [24] with Analysis of Functional NeuroImages (AFNI) software (NIMH Scientific and Statistical Computing Core; Bethesda, MD, USA). Briefly, a large ROI was drawn over contrast-enhancing regions on the ∆T1 maps in each contiguous slice, covering the entire lesion (including any macroscopic necrosis). Then, an intensity threshold was manually chosen to segment the CET (without necrosis). Each lesion was then labeled and volumes reported in microliters (μl) for each subsequent lesion.

### Statistical analysis

Median with interquartile range was reported for each lesion including tumor volume, ADC, rCBV, arterial, venous and overlap components. The normality of each distribution was evaluated using Shapiro-Wilk test. Pairwise tests of Wilcoxon-Mann-Whitney method were used to assess differences between groups for estimated variables. A *p* < 0.05 was considered to indicate a statistically significant result. Linear regression between rCBV maps and ICA-derived components were performed. Receiver-operating characteristic (ROC) analyses was performed to test the accuracy of differentiation between tumor types. The accuracy was estimates with area under the curve (AUC) as well as optimal cut-off value. We reported as well specificity and sensitivity of each parameter. All the statistics were performed using JMP Pro13 (SAS®).

## Results

**Fig. 2:**
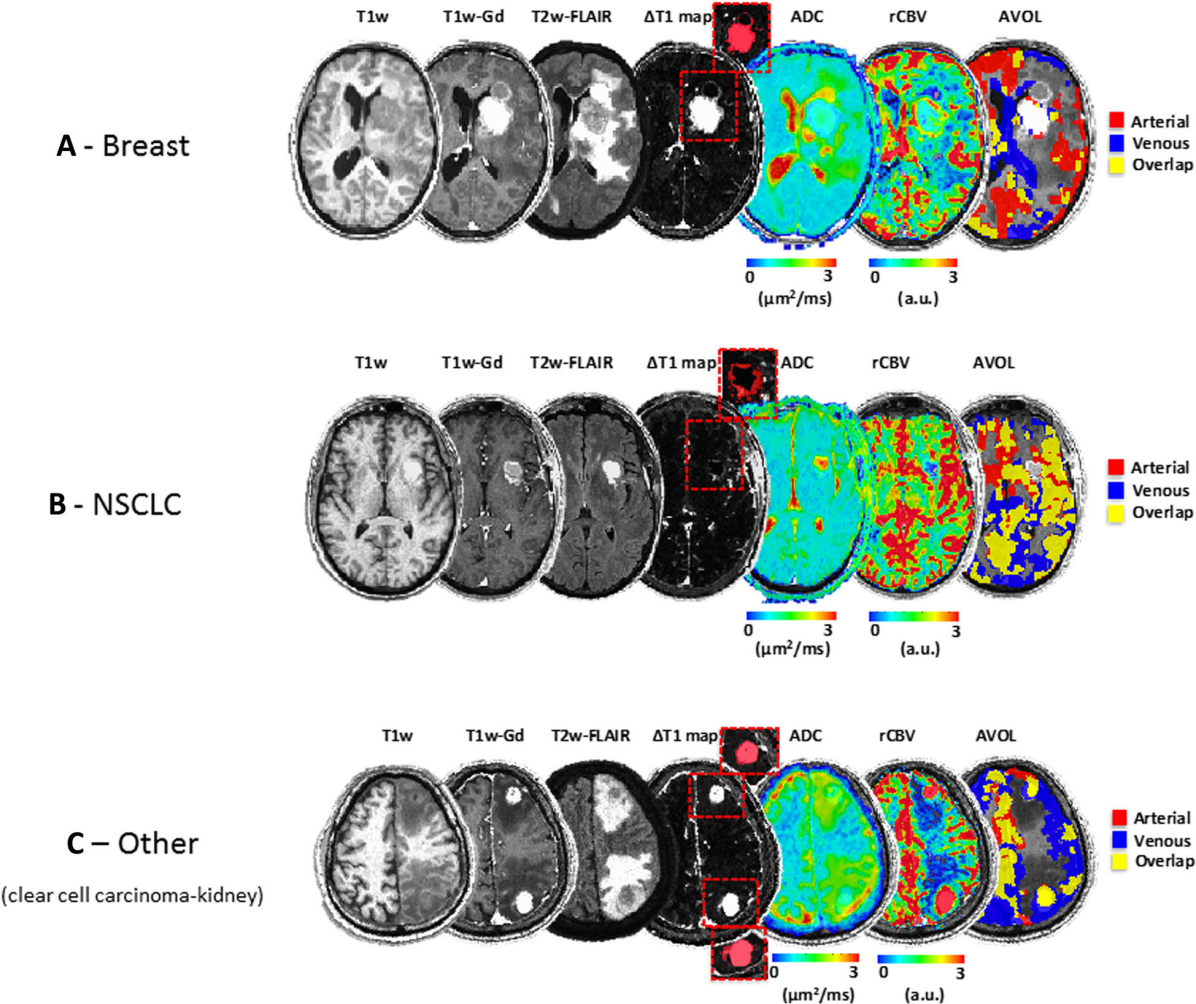
Multiparametric MRI images in patients with secondary brain metastasis from **a**) breast, **b**) NSCLC and **c**) clear cell kidney carcinoma. Pre- and post-contrast T_1_-weigthed, T_2_-weighted FLAIR, T1 subtraction (ΔT_1_ map), ADC, normalized rCBV and arterio-venous overlap (AVOL) maps for each representative patient. Inhomogeneous tumor lesions were defined and overlaid on ΔT_1_ map excluding central necrotic areas (red rectangles). T_2_-weighted FLAIR shows peri-enhancing edema on both breast and clear cell kidney carcinoma cases. ADC maps shows reduced diffusion values within solid component of the tumor (contrast enhancement) and increased diffusion in edematous component. This former region is characterized by hypoperfused blood volume (low rCBV) while solid component is mostly hyperperfused. AVOL maps are heterogeneous for both arterial and venous components while in clear cell kidney carcinoma case, tumor region is predominated by overlap map

A total of 346 lesions were examined with a median frequency of 2 lesions per patient. We analyzed 72 individual lesions from breast metastases (group range 1–3/subject), 159 from NSCLC (group range 1–4/subject) and 115 from ‘other’ group (group range 1–3/subject). Figure 2 shows anatomical (T_1_w pre- and post-contrast images, T_2_w-FLAIR and ∆T_1_ maps), diffusion (ADC) and perfusion-derived (rCBV and AVOL) images for representative patients of each group (Breast (Fig. 2a), NSCLC (Fig. 2b) and ‘other’ (Fig. 2c), respectively). All the patients clearly demonstrated blood brain barrier (BBB) disruption and contrast enhancement in all lesions. Qualitative analyses based on visual inspection revealed surrounding edema seen on T_2_w-FLAIR images, present on both patients in Fig. 2a and c and appeared in 83.9% of all lesions examined (respectively 85.1% for Breast, 86.0% for NSCLC and 80.9% for ‘other’ group). The ADC maps show higher values within tumor area (CET, red overlay), along with increased rCBV compared to NAWM (Fig. 2a, b and d). Within the CET regions, a variety of volume fractions of arterio-venous components were identified from ICA. Within areas of vasogenic edema characterized by T2 hyperintensity, we observed characteristic increased ADC and hypovascularity (low rCBV), particularly when compared to CET regions. The areas of T2 hyperintensity surrounding regions of contrast enhancement did not show any detectable arterio-venous components from ICA analysis (no AVOL observed).

### Quantification of tumor volume, ADC and rCBV metrics within enhancing lesions

**Fig. 3:**
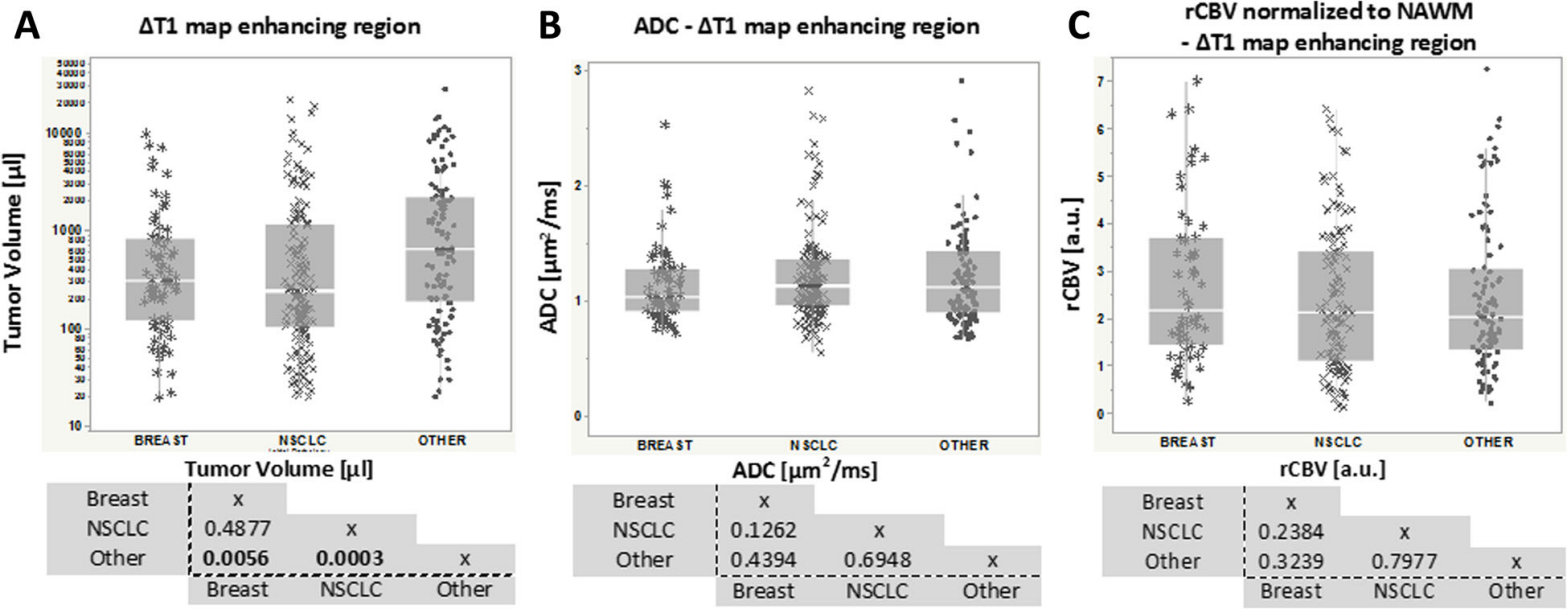
Quantitative measures of **a**) tumor volume (μL), **b**) ADC (μm2/ms) and **c**) rCBV (a.u.) for all three groups. Statistical analyses (*p* values) between groups are reported from each pair Nonparametric-Wilcoxon test. Bold text represents statistical differences (bottom), especially for tumor volume between ‘Other’ vs. Breast (*p* = 0.0056) and NSCLC groups (*p* = 0.0003)

Quantitatively, CET volumes in the non-breast, non-NSCLC ‘other’ group (median volume = 627.5 μl) were significantly larger than NSCLC (Fig. 3a; median volume = 236.0 μl, *p* = 0.0056) and primary breast cancer (median volume = 300.0 μl, *p* = 0.0003). No significant difference in median CET ADC or median CET rCBV was observed between groups (Fig. 3b and C, respectively). Statistical reports are available under each corresponding graph and numerical values (median and interquartile range) are reported per group and measurement (Table 1).

**Table 1:** Patient demographics and MRI features of different metastatic brain lesions

		**Patients,** ***N***	**Age**	**Brain Lesion,** ***N***	**Tumor Volume [μL]**	**ADC [μm** ^**2**^ **/ms]**	**rCBV**_**(normalized to NAWM,*a.u.*)**_
**Pathology**	Breast	27	56.0 (44–64)	72 (1–3)	300.0 (118.7–790.5)	1.03 (0.91–1.26)	2.14 (1.42–3.68)
	NSCLC	43	65.0 (56–71)	159 (1–4)	236.0 (104.0–1120.0)	1.12 (0.82–1.34)	2.11 (1.09–3.41)
	Other	44	62.5 (55–72)	115 (1–3)	627.53 (182.0–2121.0)	1.10 (0.88–1.42)	2.0 (1.31–3.02)
	**Total**	**114**	**60 (53.7–69.5)**	**346 (1–3)**	**318.52 (119.7–1340.9)**	**1.09 (0.91–1.34)**	**2.03 (1.23–3.39)**
					**Arterial component (%)**	**Overlap (%)**	**Venous component (%)**
			**Pathology**	Breast	27.33 (16.76–64.77)	54.13 (18.51–77.53)	54.39 (24.25–89.85)
				NSCLC	32.57 (9.01–69.04)	35.08 (11.61–59.93)	24.18 (11.33–44.60)
				Other	15.12 (4.31–33.94)	42.52 (20.99–64.14)	24.11 (7.88–50.82)
				**Total**	**23.84 (6.98–50.42)**	**40.86 (17.11–65.84)**	**29.21 (10.63–57.51)**

Patient number, age, number of brain lesion, tumor volume (μL), ADC (μm^2^/ms), normalized rCBV (*a.u.)* for each subtype of brain metastasis as well as combined cohort. Independent component analysis (ICA) derived arterial, overlap and venous components are shown for each subgroup and combined cohort. Median and interquartile ranges are reported

BOLD rows in the table represent total characteristics for the entire patient cohort examined in the current study

### Differences of AVOL components within enhancing lesions

**Fig. 4:**
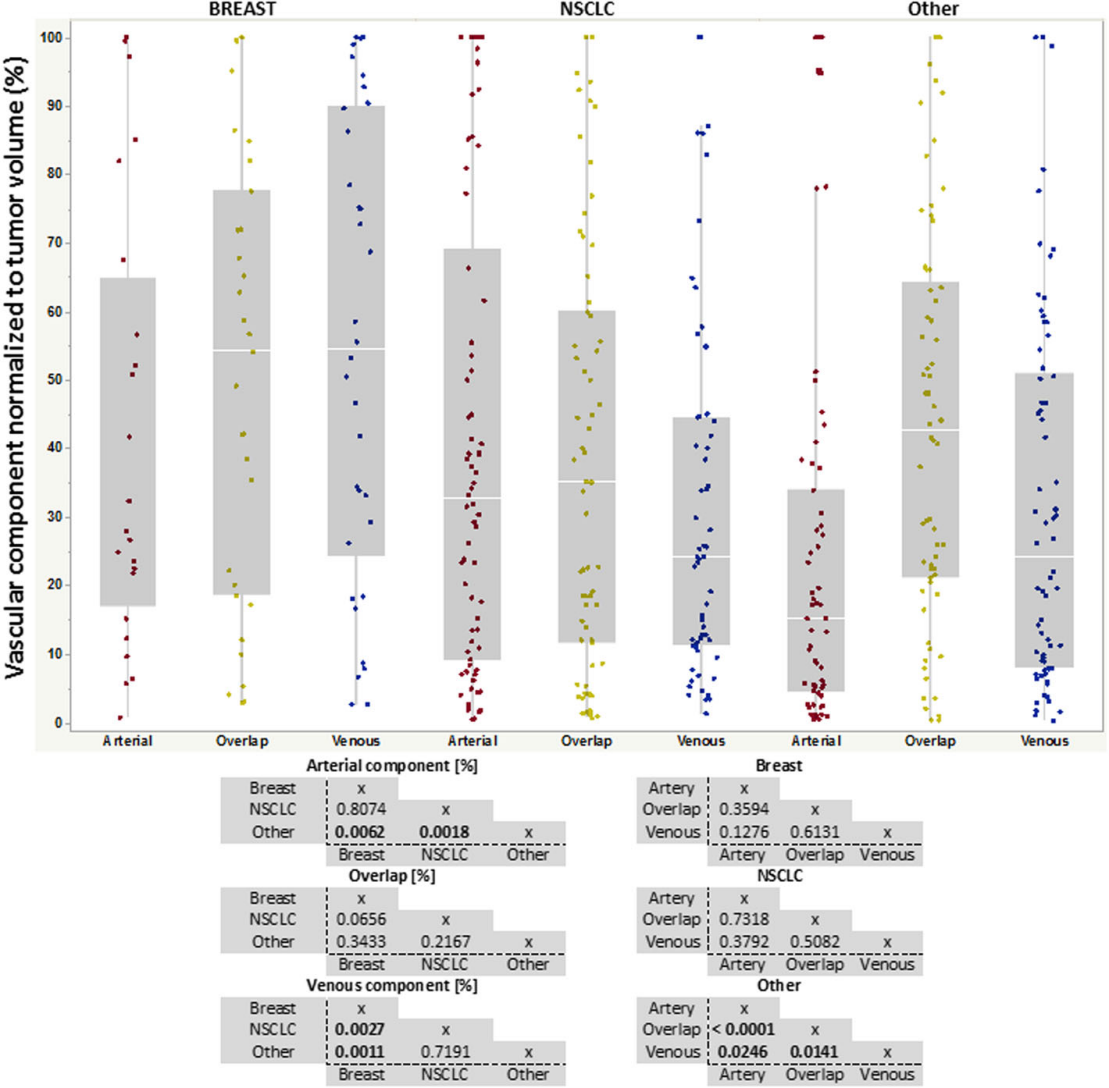
Results of arterio-venous and overlap maps from each subsequent brain metastatic group. Red, yellow and blue box plots representing respectively arterial, overlap and venous components. Statistical analyses are reported in bottom part for each component, across groups (left), as well as within each group and between components (right)

The proportion of the CET with a statistically significant “arterial” ICA component (red, Fig. 4) was significantly larger in breast metastases (median = 27.33%) and NSCLC metastases (median = 32.57%) compared with the non-breast, non-NSCLC ‘other’ group (median = 15.12%, *p* = 0.0062 and *p* = 0.0018, respectively). The proportion of the tumor with a significant “venous” component (blue) for breast brain metastases (median = 54.39%) was significantly larger when compared with NSCLC (median = 24.18%, *p* = 0.0027) and ‘other’ brain metastases (median = 24.11%, *p* = 0.0011). No difference was observed between groups for mixed proportion of vascular components (orange). While the breast and NSCLC brain metastases did not differ significantly in terms of the volume fraction of arterial, venous and overlap ICA components, the ‘other’ group contained a significant proportion of tumor with “overlap” components (mixture of arterial *and* venous components) (median = 48.66%) compared to purely arterial (*p* < 0.0001) or venous (*p* = 0.0141) components. Numerical values (median and range) are reported for each ICA component and group (Table 1).

### Temporal patterns of DSC-MRI (time to peak) in metastatic brain lesions

Results suggested no difference in TTP within arterial component, with a TTP = 7.74 s for breast (range: 7.21–9.85 s), 8.72 s for NSCLC (range: 7.60–10.50s) and 8.95 s for ‘other’ tumors (range: 6.60–11.71 s). Similarly, no differences in TTP were observed within the venous component across groups, with TTP = 12.60s for breast (range: 11.40–14.01 s), 12.67 s for NSCLC (range: 11.38–14.70s) and 12.65 s for ‘other’ group (range: 10.89–15.23 s).

### Prediction of lesion type with ROC curve analysis

We next determined by ROC analyses whether tumor types could be differentiated from each other (Table 2). Between breast and ‘other’ group, the highest accuracy was reached with venous component (AUC = 0.698, cut-off value > 68.69%). The venous component has shown the best discrimination between breast and NSCLC (AUC = 0.687, cut-off value > 46.46%). Between NSCLC and ‘other’, arterial component has shown the highest accuracy (AUC = 0.658, cut-off value < 29.26%). Similarly, arterial component was the most robust parameter to differentiate breast and NSCLC (pooled) vs. ‘other’ (AUC = 0.666, cut-off value > 20.20%).

**Table 2:** ROC curve analyses representing differentiation of Breast vs. Other, NSCLC vs. Other, Breast vs. NSCLC and pooled Breast & NSCLC groups vs. Other

Parameter	AUC	Cut-off value	Specificity	Sensitivity
Breast vs. Other				
Tumor Volume [μl]	0.62	866	45.3	81.5
ADC [μm2/ms]	0.533	1.423	25.3	91.2
rCBV [a.u.]	0.547	2.923	73.5	43.1
Arterial [%]	0.692	21.809	62.8	75
Overlap [%]	0.56	54.138	65.2	61.6
**Venous [%]**	0.698	68.688	90	44.1
NSCLC vs. Other				
Tumor Volume [μl]	0.627	266	68.7	65.3
ADC [μm2/ms]	0.513	0.972	39.2	72.9
rCBV [a.u.]	0.51	0.991	87.8	23.1
**Arterial [%]**	0.658	29.26	72.9	55.4
Overlap [%]	0.562	18.506	80.3	38.8
Venous [%]	0.518	10.514	32.6	80
Breast vs. NSCLC				
Tumor Volume [μl]	0.471	1069	25.2	83.3
ADC [μm2/ms]	0.562	0.991	66.1	47.2
rCBV [a.u.]	0.554	1.174	28.1	86.2
Arterial [%]	0.516	9.645	25.7	87.5
Overlap [%]	0.616	56.675	73.2	48.3
**Venous [%]**	0.687	46.666	78.4	58.8
Breast & NSCLC vs. Other				
Tumor Volume [μl]	0.625	556	54	68.8
ADC [μm2/ms]	0.5	1.417	26.1	84.8
rCBV [a.u.]	0.508	2.886	73.5	47.4
**Arterial [%]**	0.666	20.202	62.8	76.3
Overlap [%]	0.523	18.51	80.3	44.6
Venous [%]	0.583	72.72	92.9	33.4

Tumor volume, ADC, rCBV, ICA-derived arterial, overlap, and venous components were analyzed. For each parameter, area under curve (AUC) representing the accuracy of the measurement, cut-off value, specificity and sensitivity (expressed in %) are reported

BOLD rows in the table represent total characteristics for the entire patient cohort examined in the current study

### Correlation between DSC-MRI metrics; rCBV and AVOL maps

**Fig. 5:**
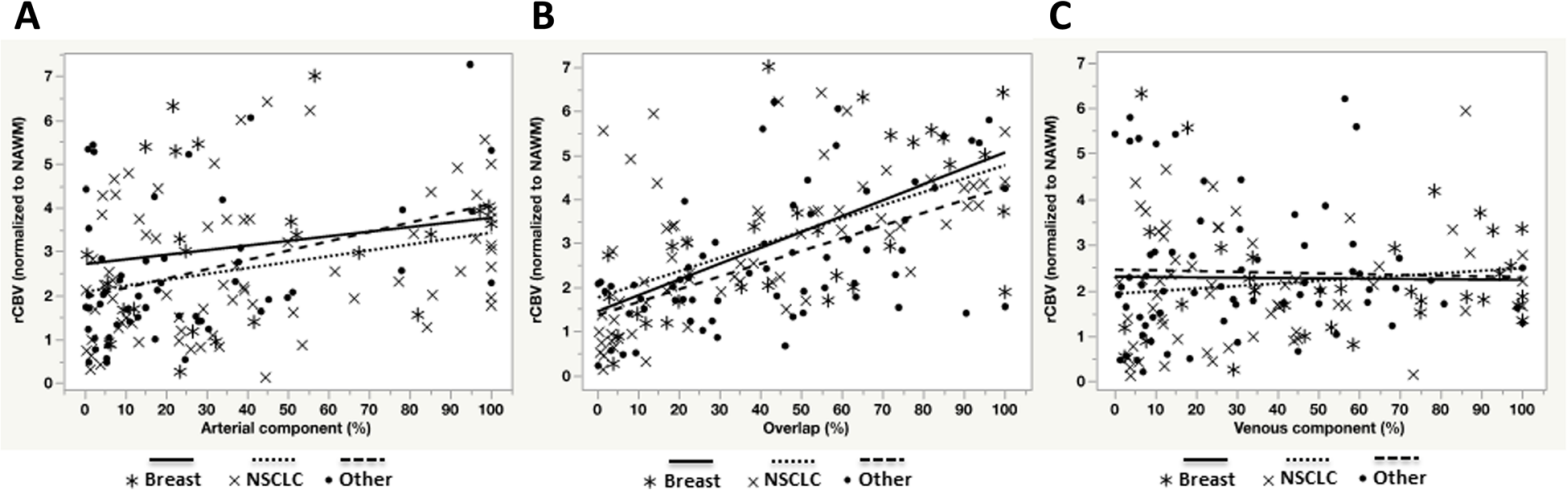
Correlation between tumor rCBV (whole ΔT1 lesion mask) and proportion of ICA-derived components. No correlation was observed between arterial component and rCBV (**a**), venous component and rCBV (**c**). The overlap proportion (**b**) significantly correlates with rCBV in each subtype of metastatic brain lesion

Lastly, we explored whether there was an inherent relationship between rCBV and ICA-derived measurements within CET. The percentage of overlap and its relationship with rCBV revealed highest linear correlation for breast: rCBV = 0.019*Arterial+ 1.551 (r = 0.68, *p* = 0.0002), NSCLC: rCBV = 0.013*Arterial+ 2.710 (r = 0.70, *p* < 0.0001) and ‘other’ group: rCBV = 0.021*Arterial+ 1.945 (r = 0.58, p < 0.0001) (Fig. 5b). No significant linear correlation was observed when comparing rCBV to arterial (Fig. 5a) or venous components (Fig. 5c). Together, this suggests measures of CET proportions exhibiting pure arterial and venous components with ICA are unique compared with traditional rCBV measures, whereas rCBV is most closely related to regions of the tumor exhibiting a mixture of arterial and venous components.

## Discussion

Although conventional MRI is often used for diagnosis and response assessment of metastatic brain tumors, MRI has traditionally been nonspecific and unable to reliably differentiate different subtypes of secondary metastatic brain lesions. While the identification of specific types of brain metastases via MRI are of limited clinical value given the primary cancer has likely already been diagnosed at the time of brain metastases identification, the specific vascular characteristics in brain metastases unique to particular primary tumor types may be of important biological significance. For example, our study suggests the proportion of CET with “venous-like” perfusion hemodynamics is higher in breast cancer compared with other tumor types, while the proportion of CET with “arterial-like” perfusion hemodynamics are substantially lower in non-breast, non-NSCLC ‘other’ tumor types. Although speculative, these differences may be explained partially by eloquent preclinical studies demonstrating differential metastatic tumor types preferring either vascular cooption or angiogenesis [25]. Specifically, breast and melanoma brain metastases have been shown to exhibit mostly vascular cooption for further continuous tumor growth [25, 26], while lung carcinoma is thought to initiate neovascularization through pro-angiogenic factors [26]. Thus, perfusion signatures that are unique to specific types of primary lesions may reflect different aspects of the tumor microenvironment or biological behavior that could be further explored or therapeutically exploited.

Results from the current study also suggest CET size of individual brain metastases from breast and NSCLC are significantly smaller than those from non-breast, non-NSCLC ‘other’ tumors. This volumes difference may also be explained by the asymptomatic screening of secondary metastatic brain lesions, detected at various stages of tumorogenesis. It is also important to note that we segmented and considered each lesion as single entity, whereas other studies analyzing the product or totality of enhancing lesions on a per patient basis. Thus, our results appear to underestimate tumor volumes reported in the literature (e.g. NSCLC) [27].

Previous investigations using diffusion and perfusion have primarily focused on glioma grading and differences between metastatic lesions versus primary brain lesions [28] and very few attempted to study brain metastases from differing primary tumor types [29–31]. We found no differences in ADC values between brain metastases from differing primary tumor types. These results were consistent with other studies, where ADC values in the current study did not differ from those previously reported in NSCLC and melanoma [32] and between lung and breast cancers [33]. Another study has found lower ADC values only in central nervous system lymphomas compared to lung, breast, melanoma, sarcoma, etc. [29]. However no significant differences were found between other tumor types, which suggest that DWI derived ADC maps, are not robust to differentiate metastatic brain lesions. In our clinic, standard perfusion measurements, including normalized rCBV, were not different between metastatic brain tumors from different primary tumor types. Our measurements of rCBV were consistent with previous reports, showing elevated rCBV (> 1.5) within solid part of the tumor in brain metastases [34]. In accord with our study, only moderate differences in rCBV have been reported among secondary brain lesions (e.g. lymphoma, breast and lung cancer metastasized to the brain) [35, 36]. In addition, Huang et al. [30], reported similar median values of normalized rCBV for the NSCLC metastases, while in breast metastases, they found more elevated rCBV values. However, the former study collects only sub-regional active tumor parts, combining 3-5 mm ROI’s and no leakage correction was performed on DSC signal, which is controversial and may have resulted in erroneous results compared with our approach.

Our findings of ICA are similar to untreated glioblastoma patients for arterial and AVOL components, but the venous component averaged ~ 29% in our study, which was lower than the 38% reported by LaViolette et al. [17]. We also confirmed that ICA-derived regions of overlap were volumetrically bigger (~ 40%) than arterial and venous tumor proportions.

During this current retrospectively study, we were not able to control or homogenize the acquisition parameters, especially slice thickness, between T1-, diffusion-, and perfusion-weighted images. Since we were not able to perfectly control the acquisition parameters, it is conceivable that differences in the timing between contrast administration and acquisition of post-contrast T1-weighted images resulted in under or overestimation of CET tumor size. Additionally, scans were performed at differing magnetic field strengths, which poses another potential limitation. However, subsequent examination indicated that only venous components were found to be significantly smaller at 3 T as compared to 1.5 T scans (18% vs. 33%, *p* < 0.005, Additional file 2: Figure S1 and Additional file 3: Figure S2), which could have potentially influenced our results. (As indicated in Additional file 3: Figure S2, if 3 T data was excluded we observed a stronger difference between venous components across tumor types). Additionally, compared with the large number of patients with brain metastases diagnosed each year, the size of the current study is relatively small and therefore results should be interpreted with caution until it can be verified in a larger, more comprehensive study.

## Conclusion

In conclusion, the current study demonstrated significant differences in vascular characteristics in brain metastases arising from specific types of primary lesions; however, we did not detect differences in conventional diffusion or perfusion characteristics between breast, NSCLC, and ‘other’ primary tumor types. A significant correlation between rCBV and ICA overlap component (volume fraction) was observed, suggesting potential sensitivity of transiting flow and ‘capillary’ fraction, and that, for all tumor types. The present results highlight the biological importance to identify abnormal vascularization (arterial, venous and overlap) in metastatic brain lesions, especially with advanced ICA post-processing approach. Finally, it is important to note that the current investigation was preliminary and genetic subgrouping and immunohistological analyses would have to be the subject of future explorations in addition to multiparametric MRI.

## Additional files

Additional file 1: **Table S1.** Histopathology of primary lesion for ‘Other’ cohort. Primary lesion site and number of case. (XLSX 8 kb)

Additional file 2: **Figure S1.** Comparison of tumor volume, ADC and rCBV in different magnetic fields. Tumor volume and rCBV were not different in 1.5 T or 3 T and that for all patient groups (blue and red, respectively). ADC value within enhancing lesion was significantly lower at 1.5 T (1.062μm^2^/ms) compared to 3 T (1.163μm^2^, *p* < 0.05) within ‘other’ group. No other valuable difference was found with standard MRI metrics. (TIF 174 kb)

Additional file 3: **Figure S2.** Comparison of arterial, venous or overlap components proportional to enhancing tumor volume at 1.5 T and 3 T. The arterial component was significantly smaller in ‘other’ group when using 1.5 T (7.59%) as compared to 3 T (17.99%, p < 0.05). We found a smaller composition of veins with 3 T (10.74%) compared to 1.5 T (30.94%, *p* < 0.005). The same pattern was seen in ‘Breast’ group at a higher proportion. We also found a median venous component composition of 72.72% with 1.5 T, while at 3 T, this value was equal to 16.62% (*p* < 0.0005). (TIF 145 kb)

## Abbreviations

∆T1: T1-weighted digital subtraction map
ADC: Apparent diffusion coefficient
AUC: Area under curve
AVOL: Arterio-venous overlap
BBB: Blood brain barrier
CET: Contrast-enhancing tumor
DSC: Dynamic susceptibility contrast
DWI: Diffusion weighted imaging
ICA: Independent component analysis
MRI: Magnetic resonance imaging
NAWM: Normal appearing white matter
NSCLC: Non-small-cell lung cancer
rCBV: Relative cerebral blood volume
ROC: Receiver operating characteristic
TR/TE: Repetition time/time to echo
TTP: Time to peak
