# Supplementary figures and images for: Probabilistic independent component analysis of dynamic susceptibility contrast perfusion MRI in metastatic brain tumors
Source: Cancer Imaging. 2019 Mar 18;19:14. doi: 10.1186/s40644-019-0201-0 (PMC6423873; doi:10.1186/s40644-019-0201-0)

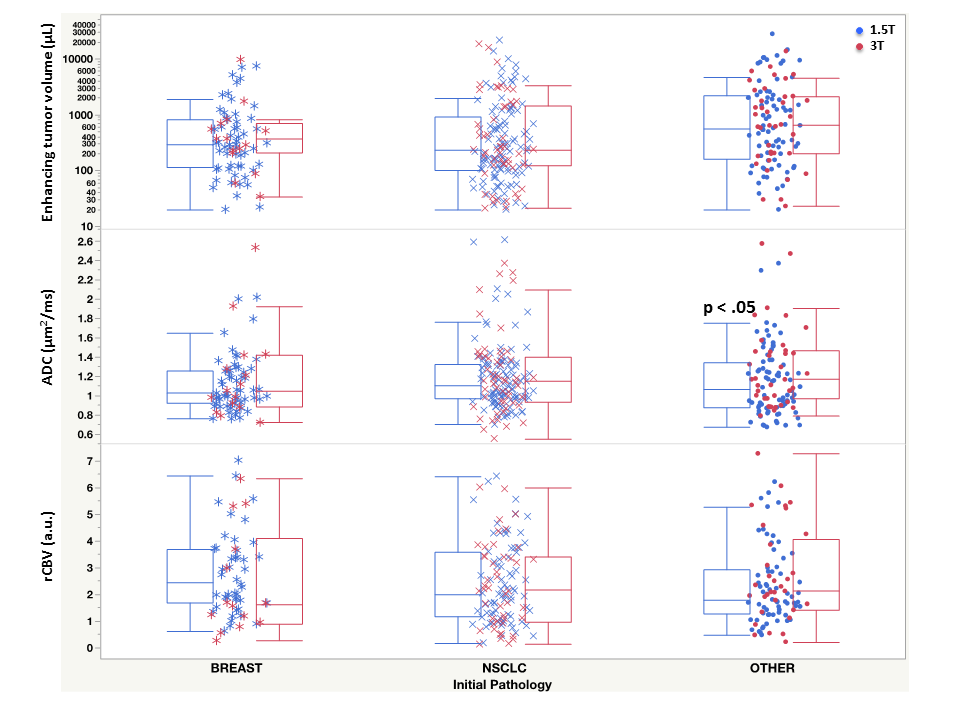

Supplement: Supplementary file 2 — Figure S1. Comparison of tumor volume, ADC and rCBV in different magnetic fields. Tumor volume and rCBV were not different in 1.5 T or 3 T and that for all patient groups (blue and red, respectively). ADC value within enhancing lesion was significantly lower at 1.5 T (1.062μm2/ms) compared to 3 T (1.163μm2, p < 0.05) within ‘other’ group. No other valuable difference was found with standard MRI metrics. (TIF 174 kb) [file 40644_2019_201_MOESM2_ESM.tif]

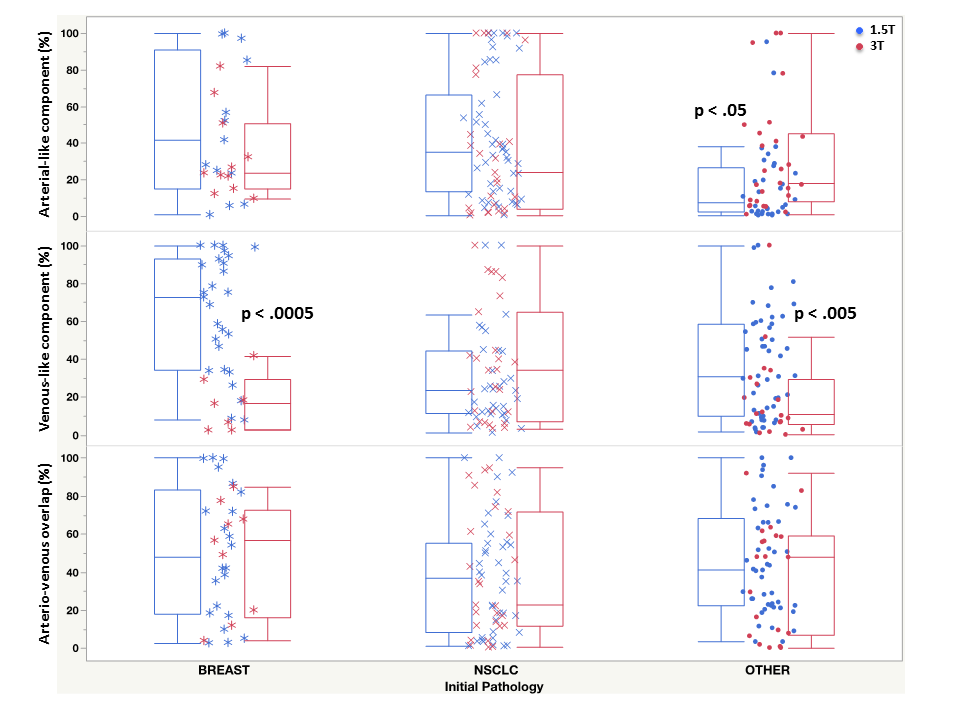

Supplement: Supplementary file 3 — Figure S2. Comparison of arterial, venous or overlap components proportional to enhancing tumor volume at 1.5 T and 3 T. The arterial component was significantly smaller in ‘other’ group when using 1.5 T (7.59%) as compared to 3 T (17.99%, p < 0.05). We found a smaller composition of veins with 3 T (10.74%) compared to 1.5 T (30.94%, p < 0.005). The same pattern was seen in ‘Breast’ group at a higher proportion. We also found a median venous component composition of 72.72% with 1.5 T, while at 3 T, this value was equal to 16.62% (p < 0.0005). (TIF 145 kb) [file 40644_2019_201_MOESM3_ESM.tif]
